# Shoulder Rotation Test: A New Test for Discriminating Between Functional and Structural Weakness

**DOI:** 10.1002/brb3.71397

**Published:** 2026-04-14

**Authors:** Takamichi Kanbayashi, Masahiro Sonoo

**Affiliations:** ^1^ Department of Neurology School of Medicine Teikyo University Tokyo Japan; ^2^ Department of Orthoptics, Faculty of Medical Technology Teikyo University Tokyo Japan

**Keywords:** external rotation, functional neurological disorder, functional weakness, internal rotation, positive sign

## Abstract

**Background:**

While many positive signs have been reported for diagnosing functional weakness (FW), additional signs are needed to enhance diagnostic accuracy. This study aims to describe and validate a new test, the “shoulder rotation test”, to discriminate between FW and structural weakness (SW).

**Methods:**

We retrospectively analyzed patients with FW or SW who presented with upper limb weakness. Inclusion criteria were that MRC scores of the shoulder internal rotation (IR) and external rotation (ER) were recorded, and that either or both were scored 4 or lower. The combination of the MRC scoring of IR and ER was named the “shoulder rotation test” and the results were classified into “weak IR with normal (MRC score of 5) ER”, “weak IR and ER”, and “normal IR with weak ER”. The former two were combined into “weak IR”.

**Results:**

In total, 29 patients with FW and 44 with SW were identified. Weak IR showed 100% sensitivity and 86% specificity for FW. Conversely, normal IR with weak ER, the complementary sign of weak IR, showed 86% sensitivity and 100% specificity for SW. Weak IR with normal ER showed 66% sensitivity and 100% specificity for FW.

**Conclusions:**

IR was preferentially weakened in FW, which is thought to be due to the preferential impairment of an “active” movement. In contrast, IR was not easily weakened in SW. The shoulder rotation test is a promising new test for discriminating between FW and SW of the upper limbs.

## Graphical abstract

We introduce the shoulder rotation test, a simple bedside test combining the MRC scoring of shoulder internal rotation (IR) and external rotation (ER), for discriminating between functional weakness (FW) and structural weakness (SW) of the upper limbs. In 29 patients with FW and 44 with SW, weak internal rotation showed 100% sensitivity and 86% specificity for FW, whereas normal IR with weak ER showed 86% sensitivity and 100% specificity for SW.

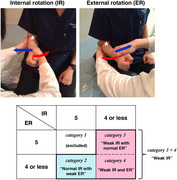

## 1 Introduction

Functional neurological disorder (FND) is a condition characterized by the involuntary manifestation of neurological symptoms despite the absence of structural abnormalities within the nervous system (Hallett et al. 2022). The prevalence of FND is estimated at 80–140 per 100,000 (Finkelstein et al. 2025), and a multi‐center study in Scotland documented that it is a common reason for referral to outpatient neurology clinics (Stone et al. 2010). Another important progress is that a consensus has been reached that FND should be diagnosed as a rule‐in diagnosis based on the positive signs that cannot be explained by structural problems of the nervous system (American Psychiatric Association 2013; Aybek and Perez 2022).

Functional weakness (FW) is one of the major phenotypes of FND (Lidstone et al. 2022). A number of positive signs for FW of the upper limb have been reported, such as drift without pronation (Daum and Aybek 2013), abduction finger sign (Tinazzi et al. 2008), and paradoxical wrist flexion (Sonoo 2021). However, it is desirable to make a diagnosis based on multiple positive signs (Stone and Aybek 2016), and therefore the description of a new positive sign is clinically valuable.

We had a preliminary impression that internal rotation (IR) of the shoulder is preferentially weakened in patients with FW, and we hypothesized that comparing the MRC scores of IR with external rotation (ER) could help discriminate between FW and structural weakness (SW) of the upper limb. We named this procedure the “shoulder rotation test”, and investigated its utility in this study.

## 2 Methods

### 2.1 Subjects

Patient data from June 2023 to July 2024 were retrospectively extracted from our in‐patient and out‐patient databases and the EMG database. Keywords used for extraction were FND for the functional group, and structural neurological disorders that may present with weakness of the upper limb for the structural group. The latter included a number of typical brain and spinal cord disorders (e.g., stroke, multiple sclerosis, motor neuron diseases, and cervical spine disorders), systemic neuropathies, and myopathies.

The inclusion criteria common to the FW and SW groups were as follows: (1) aged 18 years or older; (2) neurological examinations including manual muscle testing (MMT) were conducted by the first author (TK) or the last author (MS); (3) the Medical Research Council (MRC) scores of the IR and ER of the shoulder were recorded, and either or both were less than or equal to 4; (4) the cause of the weakness was definitely established as either FND or a certain structural disorder, the latter being confirmed by appropriate ancillary tests, such as imaging studies, neurophysiological examinations, muscle biopsy, genetic tests, or autoimmune antibody tests. The patients who could not undergo proper MMT due to severe cognitive impairment, consciousness disturbance, or severe pain were excluded.

For the FW group, we set additional inclusion criteria: (5) At least three of the following signs or findings supporting the diagnosis of FND should be present: (a) FW pattern in the Hoover test (Hoover 1908), (b) FW pattern in the abductor test (Sonoo 2004), (c) weak gluteus Maximus (Sonoo et al. 2023), (d) paradoxical wrist flexion (Sonoo 2021), (e) give‐way weakness (Stone and Aybek 2016; Daum et al. 2014), (f) normal and symmetrical reflexes despite asymmetrical weakness of the corresponding muscles, (g) normal recruitment with poor activation and no denervation potentials in needle EMG of a weak muscle, (h) other neurological signs suggesting FND such as typical functional gait. We excluded patients with functional overlay only when the comorbid SW might cause weakness of the internal or ER.

The retrospective study design was approved by the ethics committee of Teikyo University (approval number: 24–018‐2). The opt‐out method was used to obtain consent in this study.

### 2.2 Evaluation of MRC Scores of the Shoulder Internal and ERs

**FIGURE 1:**
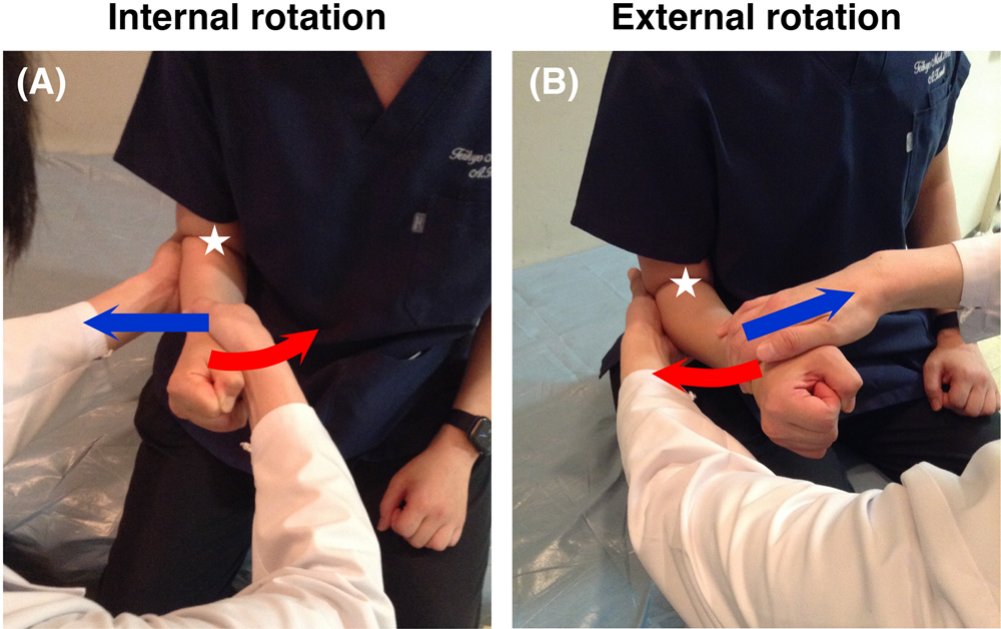
The maneuvers of the manual muscle testing for shoulder internal rotation (IR; A) and external rotation (ER; B). Red arrows indicate the direction of the force the patient applies. Blue arrows indicate the direction of the force applied by the examiner. The patient's elbow is flexed at 90° and the examiner firmly fixes the elbow against the trunk (white stars).

We evaluated MRC scores of IR and ER with the patient in a sitting position with the arm adducted towards the trunk and the elbow joint flexed at a 90° angle. During MMT, the elbow was fixed against the trunk using the examiner's non‐testing hand to enable isolated evaluation of the rotation of the shoulder joint (Figure 1). For IR, the other hand of the examiner (test hand) was placed on the medial side of the patient's forearm, and the examiner asked the patient to maintain the test position against the examiner's force externally‐rotating the arm of the patient. For ER, the test hand was placed on the lateral side of the patient's forearm, and the examiner asked the patient to maintain the test position against the examiner's internally‐rotating force.

When the patient could not maintain the test position, the MRC score was judged to be 4 or less. We did not give a score of 3 because the evaluation in a prone position was not performed. We usually evaluate the MRC score using plus or minus, for example, 4+ or 4−. However, the score was rounded to an integer number in this study. When there was a side‐difference in the muscle weakness, the scores from the weaker side were adopted. When there was no difference, the scores from the right side were used.

### 2.3 Evaluated Parameters

**FIGURE 2:**
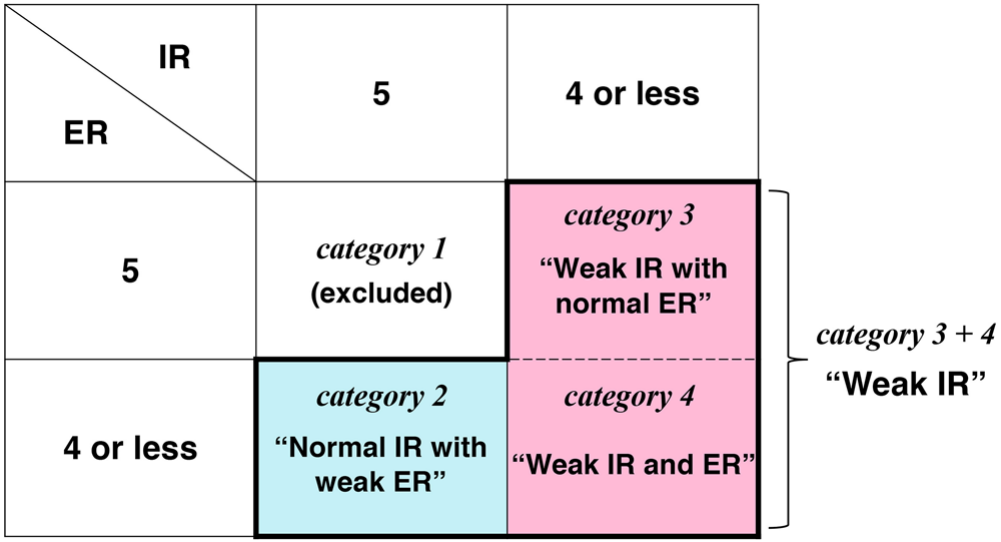
Classification according to the MRC scores of IR and ER. The categories surrounded by the thick‐lines are the study population of this study. ER, external rotation; IR, internal rotation; MRC, Medical research council.

We primarily evaluated the MRC scores of IR and ER. The results were classified into four categories according to their MRC scores (Figure 2). Patients classified as Category 1 were not included in this study so the enrolled patients consisted of Categories 2–4. Category 3 was named “weak IR with normal ER” and Category 4 was named “weak IR and ER”. The generic category combining Categories 3 and 4 was named “weak IR”. Category 2 was named “normal IR with weak ER”. The whole set of the MMT examinations of IR and ER was named the “shoulder rotation test”.

### 2.4 Statistical Analysis

The chi‐square test or Fisher's exact test were used to compare two proportions, and the Student's *t*‐test was used to compare the averages of continuous variables in different groups. The significance level was set at *p* < 0.05 and statistical analyses were performed using Microsoft Excel (Microsoft Corporation, Redmond, WA, USA).

## 3 Results

### 3.1 Enrolled Subjects

**FIGURE 3:**
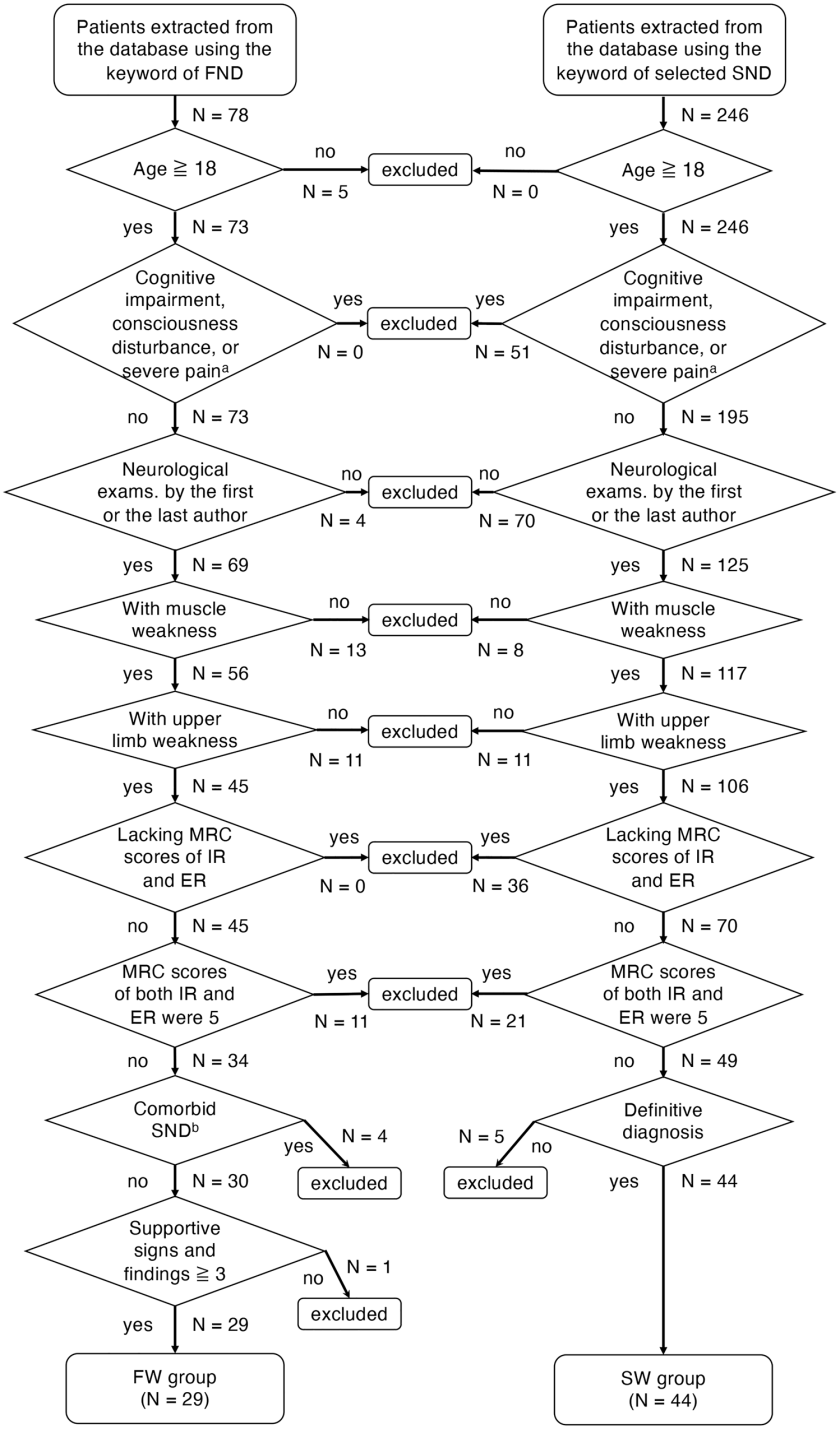
Flowchart of patient selection. (a) Patients who were not able to undergo proper MMT due to cognitive impairment, consciousness disturbance, or severe pain. (b) Comorbid SND that may affect the strength of IR or ER. ER, external rotation; FND, functional neurological disorder; FW, functional weakness; IR, internal rotation; MRC, Medical Research Council; SND, structural neurological disorder; SW, structural weakness.

Data from a total of 78 patients with FND and 246 patients with structural neurological disorder were extracted from the databases. The flowchart of the inclusion and exclusion process is shown in Figure 3. Eventually, 29 patients with FW and 44 patients with SW were identified. Their clinical characteristics, the distribution of weakness in the FW group, and the breakdown of disorders in the SW group are summarized in Table 1. The presence or absence of each positive sign in patients with FND is shown in the Table SI. The FW group was significantly younger than the SW group (*p* < 0.001). The proportion of women was significantly higher in the FW group than in the SW group (*p* < 0.05).

**TABLE 1:** Clinical characteristics of patients with FW and SW.

	FW (*n* = 29)	SW (*n* = 44)	*p* value
Age, mean ± SD (range)	44.4 ± 19.3 (20–87)	64.0 ± 15.4 (26–89)	<0.001
Men; women	9:20	25:19	<0.05
Pattern of weakness for FW			
Monoparesis			4 (14%)
Hemiparesis			9 (31%)
Tetraparesis			16 (55%)
Disorders for SW			
CNS disorders (Stroke 3, MS 1)			4
Motor neuron diseases (ALS 14, SBMA 1)			15
Spinal cord disorders (proximal CSA 9, CSM 1)			10
Neuropathies (GBS 3, CIDP 2, MMN 2)			7
Myopathies (IBM 4, dermatomyositis 1, MyD 2, SLONM 1)			8

Abbreviations: ALS, amyotrophic lateral sclerosis; CIDP, chronic inflammatory demyelinating polyradiculoneuropathy;

CNS, central nervous system; CSA, cervical spondylotic amyotrophy; CSM, cervical spondylotic myelopathy; DM, dermatomyositis; FW, functional weakness; GBS, Guillain–Barré syndrome; IBM, inclusion body myositis; MMN, multifocal motor neuropathy; MS, multiple sclerosis; MyD, myotonic dystrophy; SBMA, spinobulbar muscular atrophy; SD, standard deviation; SLONM, sporadic late‐onset nemaline myopathy; SW, structural weakness.

In the FW group, there were four patients in whom weakness in IR was revealed during the examination, despite not complaining of any upper limb symptoms. Two of them only presented with gait disturbance, one with leg weakness and gait disturbance, and one only with numbness in the lower limbs.

### 3.2 Results of the Shoulder Rotation Test

Cardinal results of the shoulder rotation test are summarized in Table 2. Weak IR showed 100% sensitivity and 86% specificity for FW. Weak IR with normal ER showed 66% sensitivity and 100% specificity for FW. Normal IR with weak ER, the complementary sign of weak IR, showed 86% sensitivity and 100% specificity for SW. The difference of frequency between the two groups was statistically significant for all three signs. All four patients (3 stroke and 1 multiple sclerosis) presenting with weakness due to pyramidal tract impairment showed the normal IR with weak ER pattern.

**TABLE 2:** Cardinal results of the shoulder rotation test.

	MRC score		FW (*n* = 29)	SW (*n* = 44)	
	IR	ER			
**Weak IR**	≤4	—	29 (100%)	6 (14%)	*p* < 0.00001
**Weak IR with normal ER**	≤4	5	19 (66%)	0 (0%)	*p* < 0.00001
Weak IR and ER	≤4	≤ 4	10 (34%)	6 (14%)	*p* < 0.05
**Normal IR with weak ER**	5	≤ 4	0 (0%)	38 (86%)	*p* < 0.00001

Abbreviations: ER, external rotation; FW, functional weakness; IR, internal rotation; MRC, Medical Research Council; SW, structural weakness.

Weak IR and ER, that is, MRC scores of 4 or less for both IR and ER, were also significantly more frequently observed in the FW group. All 10 patients with FW in this category had the same MRC score of 4 for both IR and ER. Of the six patients with SW in this category, three (2 ALS and 1 SBMA) had the same MRC score of 4 for both IR and ER. The remaining three patients had a lower MRC score for ER than for IR: IR 4 and ER 1 for a patient with ALS, and IR 4 and ER 2 for two patients with CIDP and SLONM. In this way, no patient in the SW group had a lower MRC score of IR than that of ER.

## 4 Discussion

The present results indicate that the shoulder rotation test is helpful for discriminating between FW and SW. IR was always weak in FW if either IR or ER is weak (weak IR). Accordingly, the complementary sign, normal IR with weak ER, was 100% specific to SW. Weak IR with normal ER was observed in no patients with SW, and was therefore 100% specific for FW. The SW group in this study consisted of various neurological disorders, which implies that this test is considered to be useful in actual clinical practice.

The reason why IR becomes constantly weak in FW may be common to the paradoxical wrist flexion or weak gluteus maximus (weak GM) sign (Sonoo 2021; Sonoo et al. 2023). Wrist flexion in the flexed position is interpreted by the patient as an “active” movement: In other words, it is a “highly conscious” or “purposely conducted” movement, in which the patient feels that he or she is actively exerting the relevant action with an effort. Similarly, pressing down the whole leg to the bed is interpreted as an “active”, that is, “purposely conducted” movement. Such actions seem to be preferentially impaired in FW. IR of the shoulder joint is probably interpreted as such an active movement. IR often comprises an “active” action, such as holding some other person or strongly pulling something towards oneself. In contrast, ER is not much utilized in a self‐motivated action, but often used when resisting some force pushing towards oneself. In this regard, it is an action that may be perceived as relatively passive. It is interesting that the direction of the movement is common to IR and the wrist flexion in the flexed position tested with the palm facing up, both pointing to him or herself (one's face or chest).

We can consider several reasons why IR is relatively preserved compared to ER in SW. First, IR is contributed to by many muscles innervated by different myotomes (Hislop et al. 2014), which makes it resistant to a segmental lesion. Second, IR may be intrinsically stronger than ER. Some authors have evaluated the muscle strength quantitatively using hand‐held dynamometers and found that the muscle force of IR was higher than that of ER (Andrews et al. 1996; Holt et al. 2016). Finally, ER may be preferentially involved in some pathological conditions including pyramidal weakness. All patients presenting with pyramidal tract impairment in our study showed the normal IR with weak ER pattern. Although the selectivity in pyramidal weakness has been often contradicted in recent literature (Adams et al. 1990; Thijs et al. 1998; Wiles 2017), IR belonging to muscles showing contracture in the Wernicke‐Mann posture may well be preserved in pyramidal weakness. Therefore, inverse pyramidal weakness characterized by weaker IR than ER, particularly weak IR with normal ER, may be more useful for the diagnosis of FW than global weakness, in which both IR and ER are comparably weak.

Interestingly, weak IR was observed in several patients who had no complaints of upper limb symptoms in our cohort. Some authors have reported that FW becomes apparent during neurological examinations despite the patient not complaining of any weakness in the relevant limb (Sonoo et al. 2023; Magee 1962). These facts suggest that searching for positive signs in body parts in which the patient has no complaint may help to confirm the diagnosis of FW.

This study has several limitations. First, the examiner was not blinded to the patient's diagnosis, which might have influenced the evaluation of the MRC scores. Therefore, assessing this sign with the examiner blinded to the diagnosis or quantifying it in a larger cohort would help establish its clinical utility. Second, inter‐rater reproducibility was not investigated. The MRC score is a subjective measure, and the inter‐rater variation is inevitable. The third might be the selection bias for the FW group. A gold standard or reliable diagnostic biomarkers for diagnosing FW are lacking. This limitation is common to similar studies investigating FW. In this study, we tried to guarantee the validity of the diagnosis by requiring the presence of at least three positive signs in the entry criteria. Finally, as this study was retrospective, prospective validation is required in future research.

Despite these limitations, the clear‐cut results of this study suggest that the shoulder rotation test documenting weak IR, weak IR with normal ER, or normal IR with weak ER is promising for discriminating between FW and SW.

## Author Contributions

**Takamichi Kanbayashi**: conceptualization, methodology, data curation, investigation, validation, formal analysis, funding acquisition, visualization, project administration, writing – original draft, writing – review and editing. **Masahiro Sonoo**: methodology, investigation, validation, formal analysis, supervision, funding acquisition, writing – review and editing, data curation.

## Funding

T.K. was supported by the Japan Society for the Promotion of Science, Grants‐in‐Aid for Young Scientists (JP22K15738). M.S. was supported by Grants‐in‐Aid for Scientific Research (22K07524) from the Ministry of Education, Science, Sports and Culture of Japan, and the Health and Labour Sciences Research Grant on Intractable Diseases (Neuroimmunological Diseases) from the Ministry of Health, Labour, and Welfare of Japan (20FC1030).

## Conflicts of Interest

The authors declare no conflicts of interest.

## Supporting information




**Supplementary Table S1**: brb371397‐sup‐0001‐tableS1.docx

## Data Availability

The data that support the findings of this study are available from the corresponding author upon reasonable request.
