# Supplementary material for: Shoulder Rotation Test: A New Test for Discriminating Between Functional and Structural Weakness
Source: Brain Behav. 2026 Apr 14;16(4):e71397. doi: 10.1002/brb3.71397 (PMC13080101; doi:10.1002/brb3.71397)
Supplement: Supplementary file 1 — Supplementary Table S1: brb371397‐sup‐0001‐tableS1.docx [file BRB3-16-e71397-s001.docx]

**Supplemental Table.** Presence or absence of each evaluated positive sign in patients with functional neurological disorder.

|  | Age/Sex | Hoover test | Abductor test | Weak gluteus maximus | paradoxical wrist flexion | Give-way weakness | Normal and symmetrical reflexes despite asymmetrical weakness of the corresponding muscles | Normal recruitment with poor activation and no denervation potentials in needle EMG of a weak muscle | Other neurological signs suggesting FND |
| --- | --- | --- | --- | --- | --- | --- | --- | --- | --- |
| 1 | 50, F | + | + | - | + | + | + | + | - |
| 2 | 34, F | - | - | + | + | + | - | + | - |
| 3 | 38, F | - | - | - | + | + | + | - | - |
| 4 | 26, F | - | - | - | + | + | - | + | - |
| 5 | 23, M | - | - | + | + | + | - | - | - |
| 6 | 77, F | - | - | + | + | + | - | + | - |
| 7 | 26, F | + | + | + | + | + | + | + | - |
| 8 | 23, F | - | - | - | + | + | - | + | - |
| 9 | 48, F | - | - | + | + | + | - | + | - |
| 10 | 75, F | + | + | + | + | + | - | - | - |
| 11 | 42, F | - | + | + | + | - | - | + | - |
| 12 | 59, F | - | + | + | + | + | - | + | - |
| 13 | 62, M | - | + | + | + | + | - | - | - |
| 14 | 20, F | - | - | + | - | + | - | + | - |
| 15 | 35, M | + | + | + | + | + | + | + | - |
| 16 | 51, F | + | - | + | + | + | + | + | - |
| 17 | 50, M | - | - | + | + | - | - | + | dragging gait |
| 18 | 37, F | - | - | + | + | + | - | - | - |
| 19 | 32, F | + | + | + | - | + | + | - | - |
| 20 | 36, M | - | - | + | - | + | + | - | dragging gait |
| 21 | 30, M | - | + | + | + | + | - | + | - |
| 22 | 86, M | - | - | + | - | + | - | + | - |
| 23 | 60, F | - | + | + | - | + | - | - | - |
| 24 | 87, F | - | - | + | + | + | - | + | - |
| 25 | 32, F | - | + | + | + | + | + | - | - |
| 26 | 32, M | - | - | + | + | + | - | + | - |
| 27 | 57, F | - | + | + | + | + | - | + | - |
| 28 | 38, M | - | - | - | + | + | - | + | - |
| 29 | 21, F | + | - | + | + | + | - | - | - |

“+” indicates presence of the sign. “−” indicates absence of the sign.

EMG, electromyography; F, female; FND, functional neurological disorder; M, male.
